# Longitudinal characterization of biomechanical alterations in a Parkinson's disease mouse model using three-dimensional ground reaction forces

**DOI:** 10.1016/j.prdoa.2026.100503

**Published:** 2026-09-24

**Authors:** Mengyao Ning, Jinpeng Li, Sheng Yang, Min Ye, Yi Fan, Zhendong Dai

**Affiliations:** a Institute of Bionic Structures and Materials Protection, School of Mechanical and Electrical Engineering, Nanjing University of Aeronautics and Astronautics, Nanjing, China; b Department of Pharmacology, School of Basic Medical Sciences, Nanjing Medical University, Nanjing, China; c Brain Science Center, Nanjing BenQ Hospital, Nanjing, China

**Keywords:** Mouse model, Three-dimensional GRF, Gait analysis, Quantitative analysis, Parkinson's disease

## Abstract

**Objective:**

To longitudinally characterize locomotor biomechanical changes following unilateral intrastriatal α-Syn PFF inoculation in a Parkinson's disease mouse model and explore candidate the ground reaction force (GRF) based measures for their quantitative assessment.

**Methods:**

Based on biomechanical principles, this study analyzed the GRF of mice following α-Synuclein preformed fibril (PFF) inoculation to investigate longitudinal biomechanical indicators of changes in motor function after inoculation. A unilateral intrastriatal PFF injection mouse model was established, and a three-dimensional force sensor array was developed to measure GRF in control and PFF-injected mice at 0, 1, 3, and 6 months post-modeling. Independent-samples *t*-tests, one-way analysis of variance (ANOVA), and linear regression analysis were used to evaluate GRF and gait parameters.

**Results:**

At 0-months post-modeling, no significant differences in GRF or gait parameters were observed between the control and PFF groups. At 1-month post-inoculation, a significant difference in the maximum force-vector angle of the left hindlimb was observed, together with a separate difference in right forelimb GRF. At 3-months post-modeling, the PFF group exhibited prolonged gait-cycle and stance-phase durations, accompanied by reduced left-side GRF and pronounced bilateral asymmetry. At 6-months post-modeling, interlimb asymmetry further increased, particularly in the hindlimbs, whereas some between-group differences in gait parameters became less pronounced.

**Conclusion:**

Three-dimensional GRF analysis revealed time-dependent changes in locomotor biomechanics following unilateral intrastriatal PFF inoculation. Parameters including GRF magnitude, force-vector angles, gait-cycle duration, stance-phase duration, and inter-limb asymmetry serve as candidate biomechanical markers for longitudinally characterizing motor dysfunction in mouse models of Parkinson's disease, though further validation in larger animal cohorts is required.

## Highlights

• A combined biomechanical and kinematic framework is proposed for systematic gait analysis in a PD mouse model.
• A three-dimensional force sensor array is developed to accurately capture ground reaction force-based locomotor parameters.
• Locomotor abnormalities in PD mice are quantitatively characterized by ground reaction force dynamics.
• A novel biomechanical sensing approach is introduced to investigate longitudinal differences in a PD mouse model.
• Gait cycle and stance phase duration may serve as potential biomechanical indicators of longitudinal motor function in PD.

## 1 Introduction

Parkinson's disease (PD) is a neurodegenerative disorder characterized by muscle rigidity, resting tremor, bradykinesia, and impaired balance and coordination [1]. Motor impairment in PD progresses over time and can affect mobility, balance, and independence in daily life. With population aging, PD incidence continues to increase worldwide. According to the *China Parkinson's Disease Report 2025*, China has more than 5 million PD patients, accounting for 43.14% of global cases [2]. Su et al. [3], based on the *Global Burden of Disease Study 2021*, predicted that the global PD population will reach 25.2 million by 2050, highlighting PD as a major public health challenge [4].

PD progression includes the prodromal, mild, moderate, and severe stages and is closely associated with α-Synuclein (α-Syn) aggregation [5]. Early motor abnormalities are subtle and easily overlooked, whereas advanced disease leads to severe motor dysfunction and long-term dependence. Previous studies have reported abnormalities in muscle activation, kinematics, force generation, and postural control in PD patients [6], [7]. These findings support exploratory research employing sensitive quantitative metrics to characterize motor dysfunction and its progression.

Gait reflects the interaction among the nervous system, musculoskeletal system, and ground reaction forces (GRF). Neurological disorders alter gait patterns and force characteristics, making gait analysis valuable for evaluating PD-related motor dysfunction [8], [9]. Kenneth et al. [10] first investigated locomotion speed and step frequency in rats. Amende et al. [11] demonstrated that gait alterations in PD and Huntington's disease models reflect neural pathway damage. Chuang et al. [12] evaluated gait changes in mice injected with α-Synuclein preformed fibrils (PFFs) using the Cat-Walk system. Klein et al. [13] reported improved gait stability after striatal dopamine grafting. Goldberg et al. [14] identified abnormalities in gait parameters in MPTP-induced PFF-injected mice, and Beare et al. [15] distinguished normal mice from spinal cord injury models using gait analysis. These findings suggest that gait parameters may serve as sensitive biomarkers for neurological disorders.

Clinically, the diagnosis of PD is primarily based on bradykinesia accompanied by resting tremor, rigidity, or both [16], [17]. Although GRF has not been incorporated into the clinical diagnostic or screening criteria for PD, it can objectively characterize foot–ground interactions under neural control and may reveal motor abnormalities that are difficult to detect through visual observation alone [18], [19]. Previous studies have shown that patients with PD exhibit biomechanical alterations, including reduced vertical GRF (vGRF) peaks, prolonged gait-cycle duration, altered stance/swing-phase proportions, and abnormalities in foot loading and interlimb symmetry, suggesting that these measures may have potential value for disease discrimination and severity assessment [20], [21], [22]. In addition, anterior–posterior (AP) and mediolateral (ML) GRF and center-of-pressure (CoP) measures can reflect braking and propulsion capacity, lateral weight transfer, and dynamic postural control, with abnormalities associated with gait initiation deficits, freezing of gait, and fall risk [23], [24], [25]. However, GRF is influenced by walking speed, and these gait-related measures remain investigational and cannot be considered independent diagnostic biomarkers for PD [26].

Moreover, multidimensional force measurement remains limited by cost and equipment availability. Existing human studies have mainly focused on vGRF or plantar pressure during cross-sectional walking tasks, whereas three-dimensional GRF analysis has rarely been applied to PD animal models with a defined pathological onset. Therefore, we developed a high-precision three-dimensional force sensor array and employed a unilateral intrastriatal α-Syn PFF model. This model provides a temporally defined initiation of α-Syn aggregation and a controlled lateralized perturbation, allowing within-animal comparisons of left- and right-limb biomechanics. This study aims to longitudinally characterize GRF and gait alterations in PFF-injected mice and to explore candidate biomechanical markers for assessing pathological progression in this mouse model of Parkinson's disease. It does not aim to directly map specific post-injection time points to the pathological stages or diagnostic categories of human PD.

## 2 Materials and methods

Animal models are widely used to investigate the pathogenesis and functional consequences of neurodegenerative diseases. In this study, α-Synuclein preformed fibrils (PFFs) were used to establish a longitudinal synucleinopathy model. This model was selected because exogenous PFFs can induce endogenous α-Syn aggregation, thereby allowing locomotor changes to be examined over defined post-inoculation intervals. We previously established a preparation and quality-control procedure for recombinant human wild-type α-Syn PFFs and independently characterized the pathological and behavioral phenotypes of a closely related unilateral intrastriatal PFF injection model [27], [28]. At 0, 1, 3 and 6 months after injection, we measured the ground reaction forces of control and PFF-injected mice walking through a constrained walkway using a three-dimensional force sensor array. We then quantified body-weight-normalized GRF, gait phases, and gait cycles of all four limbs and analyzed the angle between the three-dimensional force vector and the ground normal to characterize longitudinal biomechanical differences between control and PFF-injected mice.

### 2.1 Experimental animals

In this study, we used male C57BL/6 J mice aged 10–12 weeks, with body weights ranging from 24 to 28 g. The Experimental Animal Center of Nanjing Medical University provided all the animals. We housed the mice at a controlled temperature of 24 ± 2 °C and maintained relative humidity between 40% and 70%. They had ad libitum access to a standard pellet diet and water. We maintained a 12-h light/12-h dark cycle throughout the experiment. The Institutional Animal Care and Use Committee of Nanjing Medical University approved all procedures.

### 2.2 Α-Syn PFF preparation and PD model establishment

Recombinant human wild-type α-Syn was prepared and purified according to our previously established protocol [27]. Bacterial endotoxin was removed using an endotoxin-removal kit, and the endotoxin concentration was confirmed to be <0.12 EU/mL. Purified monomeric α-Syn was adjusted to 5 mg/mL in sterile phosphate-buffered saline (PBS) and incubated at 37 °C with continuous shaking at 1000 rpm for 7 days to induce fibril formation. In our established protocol, PFF formation was verified by Thioflavin T fluorescence, SDS-PAGE, and electron microscopy. After sonication, the majority of fibrils were < 50 nm in length, with a mean length of 41.96 ± 0.80 nm. Following established best-practice recommendations for α-Syn PFFs [29], mature PFFs were divided into single-use aliquots and stored at −80 °C to minimize repeated freeze-thaw cycles. On the day of use, one aliquot was thawed at room temperature, diluted to 2 μg/μL in sterile PBS, and sonicated immediately before use (six sonication cycles; total 10 s, 1 s on/1 s off) [27].

Six mice were randomly selected; three received a unilateral intrastriatal injection of α-Syn PFFs (PFF group), while the other three received an injection of normal saline (control group). Under 4% chloral hydrate anesthesia (0.01 mL/g), mice were fixed in a stereotaxic frame after scalp preparation and skull exposure. Using bregma as the reference point, α-Syn PFF (8 μg, 2 μg/μL; 4 μL) was injected into the right striatum at AP +0.2 mm, ML +2.0 mm, and DV −2.6 mm to induce the model. Three control mice received 2.5 μL saline at 0.4 μL/min. After injection, the needle was retained for 5 min and withdrawn slowly over 5 min. All procedures were performed under sterile conditions. Following wound suturing and disinfection, mice recovered on a heating pad before returning to standard housing.

The unilateral right-striatal paradigm was chosen deliberately because it provides a spatially defined perturbation of the nigrostriatal system while preserving a within-animal left-right functional contrast for biomechanical analysis. In addition, we conducted time-matched neuropathological assessments on the experimental groups to monitor α-Syn pathology and the integrity of the nigrostriatal pathway, thereby providing pathological support for this study. Notably, these neuropathological markers were not the primary quantitative endpoints of this biomechanical study, nor did we perform individual-level correlation analyses between pathological load and GRF or gait parameters. In an independent cohort from our laboratory using the same PFF preparation procedure and a closely related unilateral intrastriatal paradigm, increased phosphorylated α-Syn was detectable at 3-months before significant loss of TH-positive nigral neurons, whereas by 6-months increased phosphorylated α-Syn, reduced TH in the ipsilateral midbrain and striatum, loss of TH-positive neurons in the substantia nigra pars compacta, and motor deficits were evident [28]. These published data provide additional pathological context and independent validation of the temporal profile observed in our experiments.

### 2.3 Experimental setup

The experimental system consisted of a 3 × 8 array of three-dimensional force sensors (NBIT, Nanjing, China) with a 1.5 N range, 0.2 mN resolution, and 100 Hz sampling rate. Two high-speed cameras (Prime 41, OptiTrack, USA) were positioned at 45° above the array and recorded at 200 frames/s. Motion capture was performed using the Motive system (OptiTrack, USA). An acrylic channel constrained mice to straight-line locomotion. Force signals were acquired using an SCXI data acquisition system (NI, Inc., USA) and recorded in LabVIEW. Force and video data were synchronized.

### 2.4 Experimental procedure

We built an experimental system consisting of a force platform and cameras. Before testing, mice were allowed to adapt to the channel and received straight-line walking training. As shown in Fig. 1A, a schematic of the experimental setup used in this study illustrates the force platform and the camera position for recording mouse locomotion. The channel width was adjusted to permit passage of a single mouse. A quiet, dim environment was maintained to reduce behavioral interference. For each trial, a mouse was placed at the channel entrance facing the +Y direction. Tail stimulation with a soft brush encouraged forward movement. The channel was cleaned with 75% medical alcohol after each trial to remove odor cues. Trials were excluded if the mouse stopped during locomotion.

**Fig. 1:**
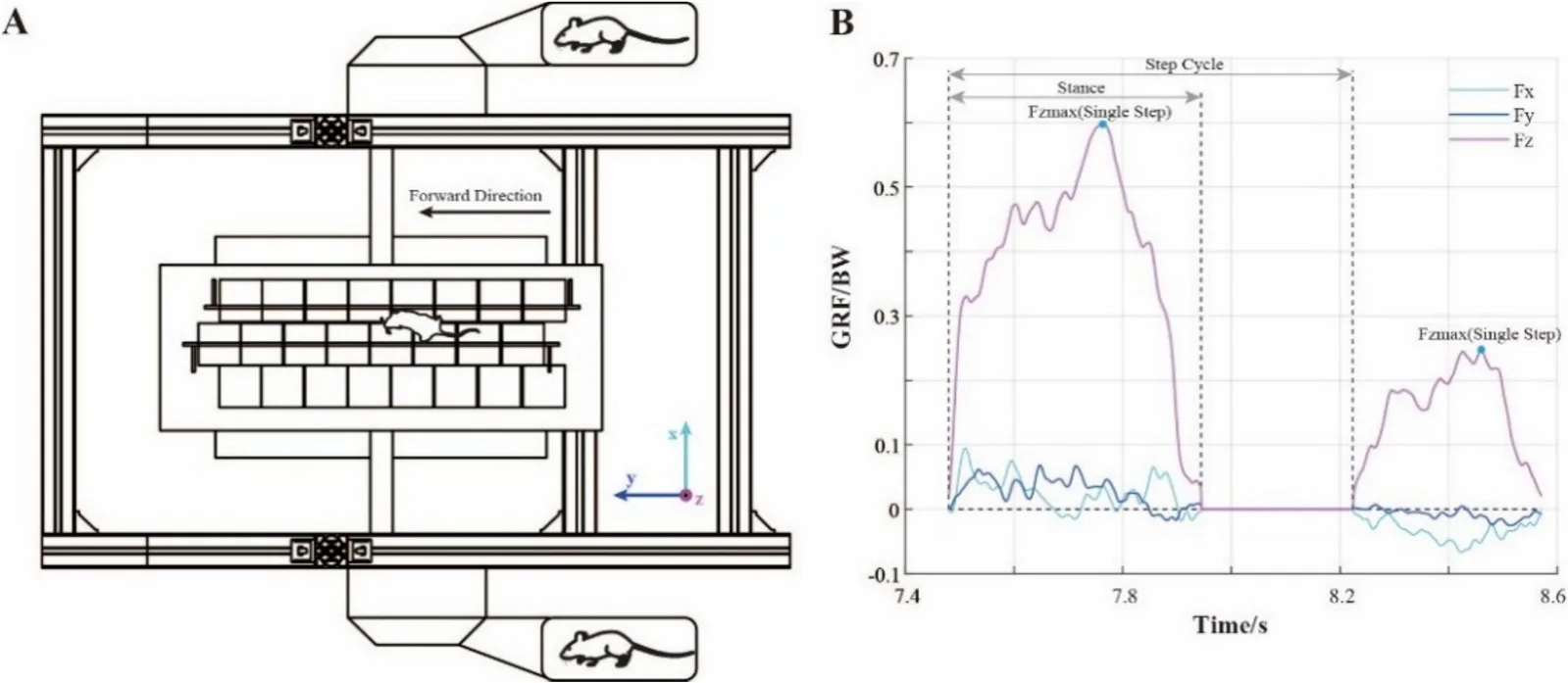
Experimental method for measuring ground reaction forces and gait parameters in mice during straight-line locomotion. A. Schematic illustration of the experimental setup. B. Representative example of three-dimensional force data recorded by a single sensor during mouse locomotion.

The left forelimb, left hindlimb, right forelimb, and right hindlimb were defined as LF, LH, RF, and RH. A gait cycle was defined as the interval between two consecutive contacts of the same limb. Mice completed at least two consecutive gait cycles on the sensor array during each trial. High-speed video recordings were used to verify gait validity. Trials were discarded when any limb contacted the sensor array more than once within a gait cycle. At each time point, each mouse completed five valid trials, and body weight was recorded for GRF normalization and subsequent analysis.

## 3 Results and analysis

### 3.1 Three-dimensional ground reaction forces

To investigate locomotor biomechanics and gait characteristics in normal and PFF-injected mice, we normalized ground reaction forces to body weight. *F*_x_, *F*_y_, and *F*_z_ represented mediolateral, anteroposterior, and normal forces, respectively. We analyzed the data from three aspects: (1) We plotted time-series curves of *F*_x_, *F*_y_, *F*_z_ for each sensor and extracted the maximum vertical GRF (*F*_zmax_) during consecutive gait cycles. Synchronized video recordings enabled assignment of each force peak to the corresponding limb. We defined the stance phase as the period from initial paw contact to paw-off and the swing phase as the interval from paw-off to the next contact. GRF and gait parameters were used to quantify locomotor performance. (2) We constructed a spatial representation of GRF during the stance phase. A vector surface was generated using *F*_x_, *F*_y_ and *F*_z_ as the three components of the force vector. GRF variation, pressure-angle changes, and the maximum projection angles of the force-vector were then characterized. (3) We calculated the maximum angles in each direction from the stance-phase force vector data and used these parameters to evaluate the stability of limb loading in different directions.

These parameters were used to longitudinally characterize changes in locomotor function and limb balance control between PFF-injected and control mice and to explore their potential utility as biomechanical measures. Using IBM SPSS 25, we conducted statistical analyses. Independent-samples *t*-tests were used for comparisons between two groups. One-way analysis of variance (ANOVA) was applied to comparisons among multiple groups or time points. Linear regression analysis assessed relationships between continuous variables. Statistical significance was defined as *p* < 0.05. All data are presented as mean ± standard deviation (Mean ± SD).

### 3.2 Ground reaction forces of left and right limbs

Throughout the experiment, no significant difference in vertical ground reaction force was observed between the left and right limbs of control mice. We therefore compared the kinetic characteristics of control mice (PBS) and PFF-injected mice (PFF). At 0-months post-modeling (Fig. 2A), no significant difference in GRF was detected between the two groups (Left: 0.51 ± 0.07 vs. 0.54 ± 0.10, *p* > 0.05; Right: 0.54 ± 0.10 vs. 0.54 ± 0.11, *p* > 0.05; *n* = 3). At 1-month post-modeling (Fig. 2B), no significant difference was observed between the left and right limb GRF in PFF-injected mice or between control and PFF-injected mice (Left: 0.55 ± 0.11 vs. 0.51 ± 0.11, *p* > 0.05; Right: 0.53 ± 0.08 vs. 0.53 ± 0.11, *p* > 0.05), indicating no significant difference in limb GRF between the two groups at this time point. At 3-months post-modeling (Fig. 2C), left-limb GRF in PFF-injected mice was lower than right-limb GRF, but the difference was borderline (0.51 ± 0.10 vs. 0.55 ± 0.11, *p* = 0.05). Left-limb GRF was also lower than that of control mice (0.51 ± 0.10 vs. 0.57 ± 0.12, *p* = 0.002). No significant difference was observed in the right-limb GRF between groups (0.58 ± 0.08 vs. 0.55 ± 0.11, *p* > 0.05). These results indicated that group differences in GRF became detectable at 3-months and were more evident on the left side. At 6-months post-modeling (Fig. 2D), left-right GRF asymmetry further increased in PFF-injected mice (0.46 ± 0.14 vs. 0.54 ± 0.14, *p* = 0.0004). The left-limb GRF remained significantly lower than that of control mice (0.46 ± 0.14 vs. 0.52 ± 0.13, *p* = 0.006), whereas the right-limb GRF showed no significant difference between groups (0.53 ± 0.13 vs. 0.54 ± 0.14, *p* > 0.05). These findings demonstrate a time-dependent increase in left-right GRF asymmetry, with a predominant reduction in left-side loading in PFF-injected mice.

**Fig. 2:**
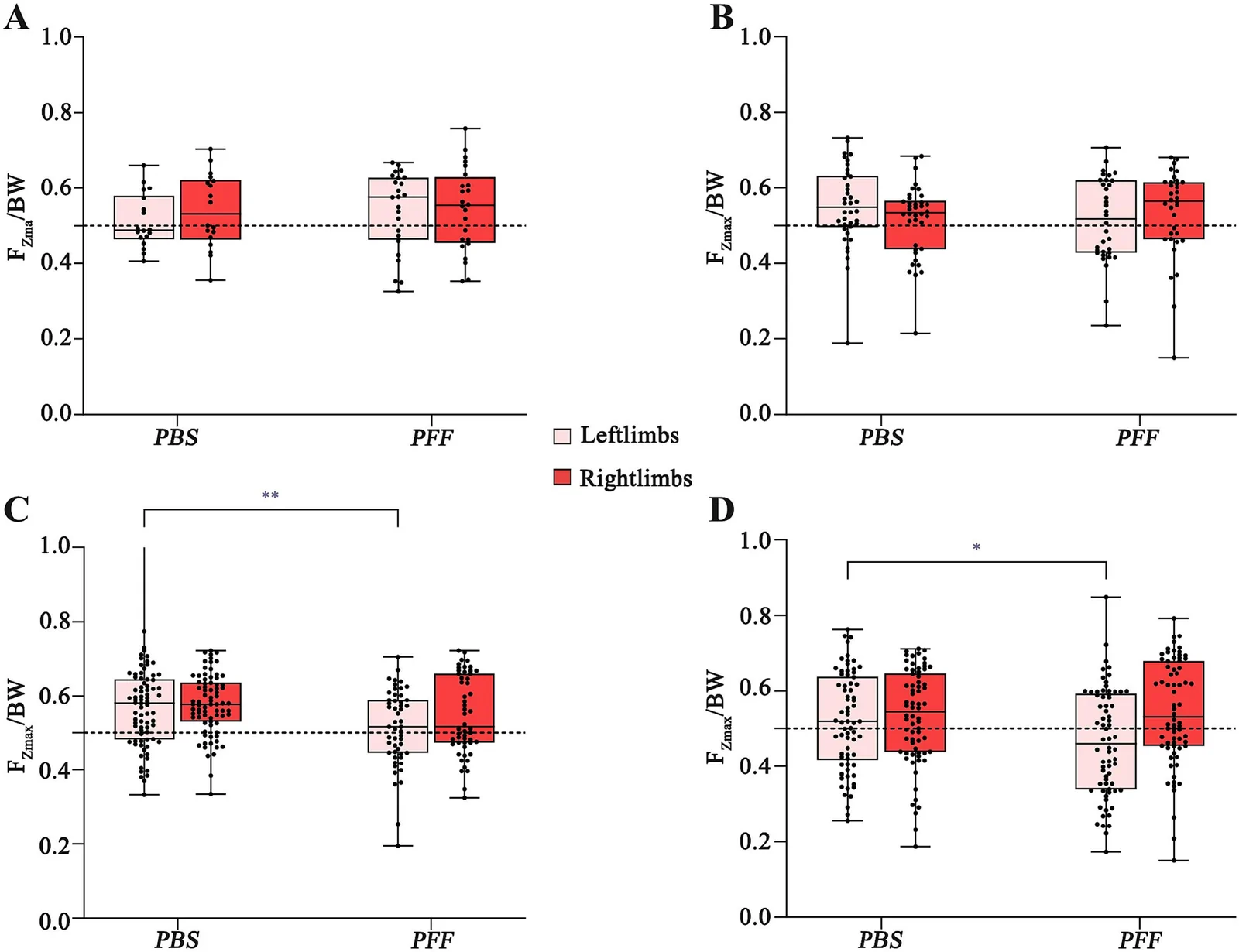
Maximum body-weight-normalized vertical GRF on the left and right feet of control and PFF-injected mice at different time. A. 0-months. B. 1-month. C. 3-months. D. 6-months (* *p* < 0.05, * * *p* < 0.01).

### 3.3 Ground reaction forces of four limbs

To further evaluate longitudinal changes in limb kinetics, four-limb ground reaction forces in control and PFF-injected mice were statistically analyzed at different post-modeling time points. As shown in Fig. 3A, no significant differences were observed between the two groups at baseline (0 months post-modeling). At 1-month post-modeling (Fig. 3B), the ground reaction forces of the left forelimb (LF), right hindlimb (RH), and left hindlimb (LH) in PFF-injected mice showed slight decreases without statistical significance. However, a significant difference was observed in the right forelimb (RF) between control and PFF-injected mice (0.54 ± 0.11 vs. 0.61 ± 0.04, *p* = 0.03). At 3-months post-modeling (Fig. 3C), the ground reaction forces of the right hindlimb and left hindlimb were lower in PFF-injected mice (RH: 0.45 ± 0.09; LH: 0.47 ± 0.08) than in control mice (RH: 0.52 ± 0.08; LH: 0.53 ± 0.07), and these differences were statistically significant (*p* = 0.0036 < 0.01). At 6 months post-modeling (Fig. 3D), a significant difference in ground reaction force was observed only in the left hindlimb between the control and PFF-injected groups (0.44 ± 0.10 vs. 0.36 ± 0.10, *p* = 0.0163). Together with the earlier findings, these results indicate a persistent reduction in left hindlimb ground reaction force during the 3–6 months post-modeling period following PFF inoculation, consistent with sustained alterations in limb loading and locomotor biomechanics.

**Fig. 3:**
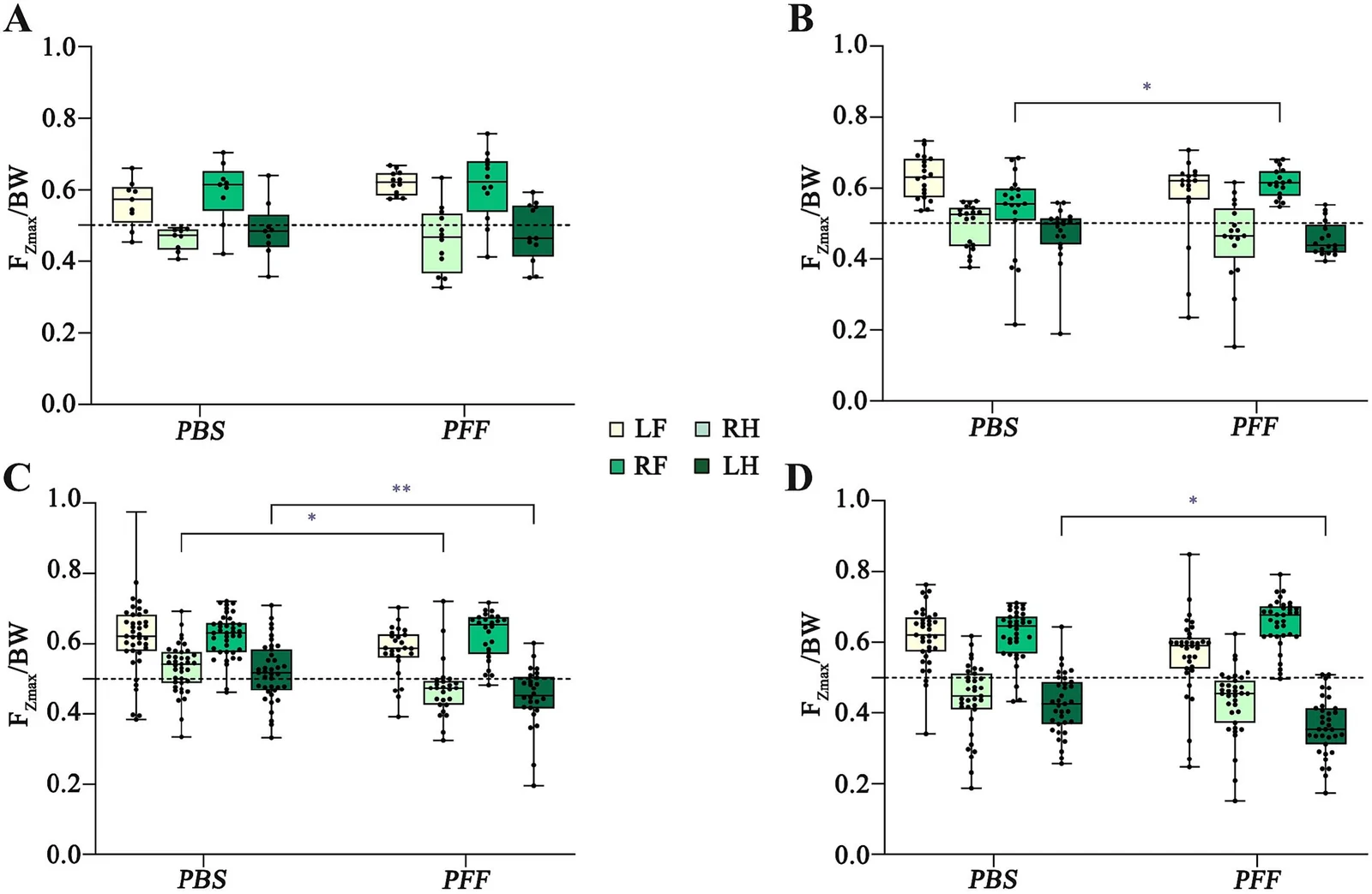
Maximum body-weight-normalized vertical GRF of the four limbs in control and PFF-injected mice at different time points. A. 0-months. B. 1-month. C. 3-months. D. 6-months (* *p* < 0.05, * * *p* < 0.01).

### 3.4 Gait cycle and stance phase duration of left and right limbs

Based on the analysis of left–right limb ground reaction forces, we further compared gait cycle and stance phase duration between control and PFF-injected mice to assess longitudinal changes in motor control following PFF inoculation. As shown in Fig. 4, gait cycle duration in both groups showed an initial increase followed by a decrease from 0 to 6-months post-modeling. At 0- months and 1-month post-modeling (Fig. 4A, B), no significant differences in gait-cycle duration were observed between the control and PFF-injected groups in either the left or right limbs, indicating that locomotor timing was not detectably altered at these time points. At 3-months post-modeling (Fig. 4C), significant differences in gait-cycle duration were observed in both the left and right limbs between the two groups (Left: 0.64 ± 0.25 s vs. 0.96 ± 0.32 s, *p* = 0.0005; Right: 0.67 ± 0.33 s vs. 0.97 ± 0.28 s, *p* = 0.001), with PFF-injected mice exhibiting prolonged gait cycles, indicating altered locomotor timing. At 6-months post-modeling (Fig. 4D), the significant between-group differences in gait-cycle duration were no longer observed, which may reflect adaptive changes in locomotor strategy. However, this possibility cannot be confirmed from the present biomechanical measurements alone.

**Fig. 4:**
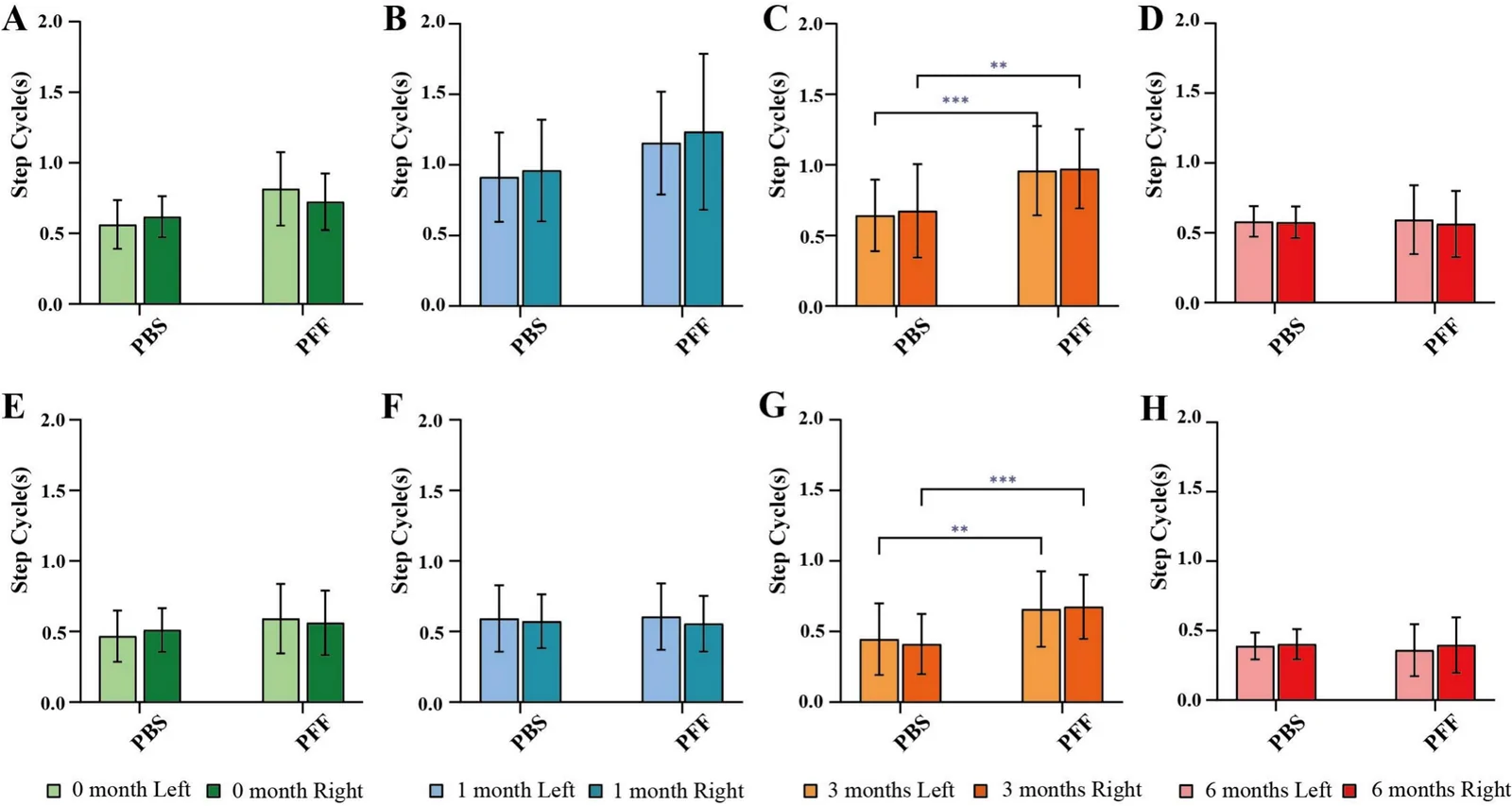
A–D: Gait cycle duration of control and PFF-injected mice at 0, 1, 3 and 6 months after modeling. E–H: Stance duration of control and PFF-injected mice at 0, 1, 3 and 6 months after modeling (* *p* < 0.05, * * *p* < 0.01, * * *p* < 0.001).

In contrast, the stance-phase duration of the lateral limbs showed a distinct temporal pattern. It remained stable in control mice but changed from an increasing trend during the early stage to a marked decrease at 6-months post-modeling in PFF-injected mice. At 0-months and 1-month post-modeling (Fig. 4E, F), no significant differences were observed between control and PFF-injected mice, indicating that no significant alteration in limb function was detectable during the early post-inoculation period. At 3-months post-modeling (Fig. 4G), significant differences in stance phase duration were observed between groups. On the left side, values increased from 0.44 ± 0.26 s to 0.66 ± 0.27 s (*p* = 0.0041), while on the right side, values increased from 0.41 ± 0.26 s to 0.68 ± 0.23 s (*p* = 0.0004). These findings are consistent with altered bilateral locomotor timing and prolonged foot-ground contact time in PFF-injected mice. At 6-months post-modeling (Fig. 4H), intergroup differences decreased and no significant differences remained, suggesting increased similarity in limb movement patterns. Overall, the difference in stance-phase duration between the two groups was most pronounced at 3 months post-modeling, whereas the between-group difference was markedly reduced at 6 months. The between-group difference was smaller at 6 months, but the present measurements cannot determine whether this pattern reflects compensatory adaptation, temporal variability, or a change in locomotor strategy.

### 3.5 Maximum angles of limb ground reaction force vectors

To quantify locomotor balance, we calculated the maximum angles between limb GRF vectors and the *Z*-axis. As shown in Fig. 5A and E, no significant differences were observed between control and PFF-injected mice at 0 months, indicating that there were no apparent disparities in these force vector parameters at that time. At 1-month (Fig. 5B, F), significant differences in *α*ₙ and *α*_p_ were detected in the X–Z plane, mainly in the left hindlimb. Specifically, *α*ₙ decreased from 27.13° ± 11.55° in control mice to 17.44° ± 8.51° in PFF-injected mice (*p* = 0.009), whereas no significant differences were observed in the Y–Z plane. These results indicate that a difference in unilateral motor function emerged at this time point. At 3-months (Fig. 5C, G), significant differences in force-vector angles were observed in the left forelimb (*α*ₙ, *p* = 0.002), left hindlimb (*α*_p_, *p* = 0.03), and right forelimb (*α*_p_, *p* = 0.004). The left forelimb exhibited the most significant changes in vector parameters, indicating the presence of left-predominant biomechanical alterations at this stage. At 6-months post-modeling (Fig. 5D, H), no significant differences in force-vector angles were observed between the two groups.

**Fig. 5:**
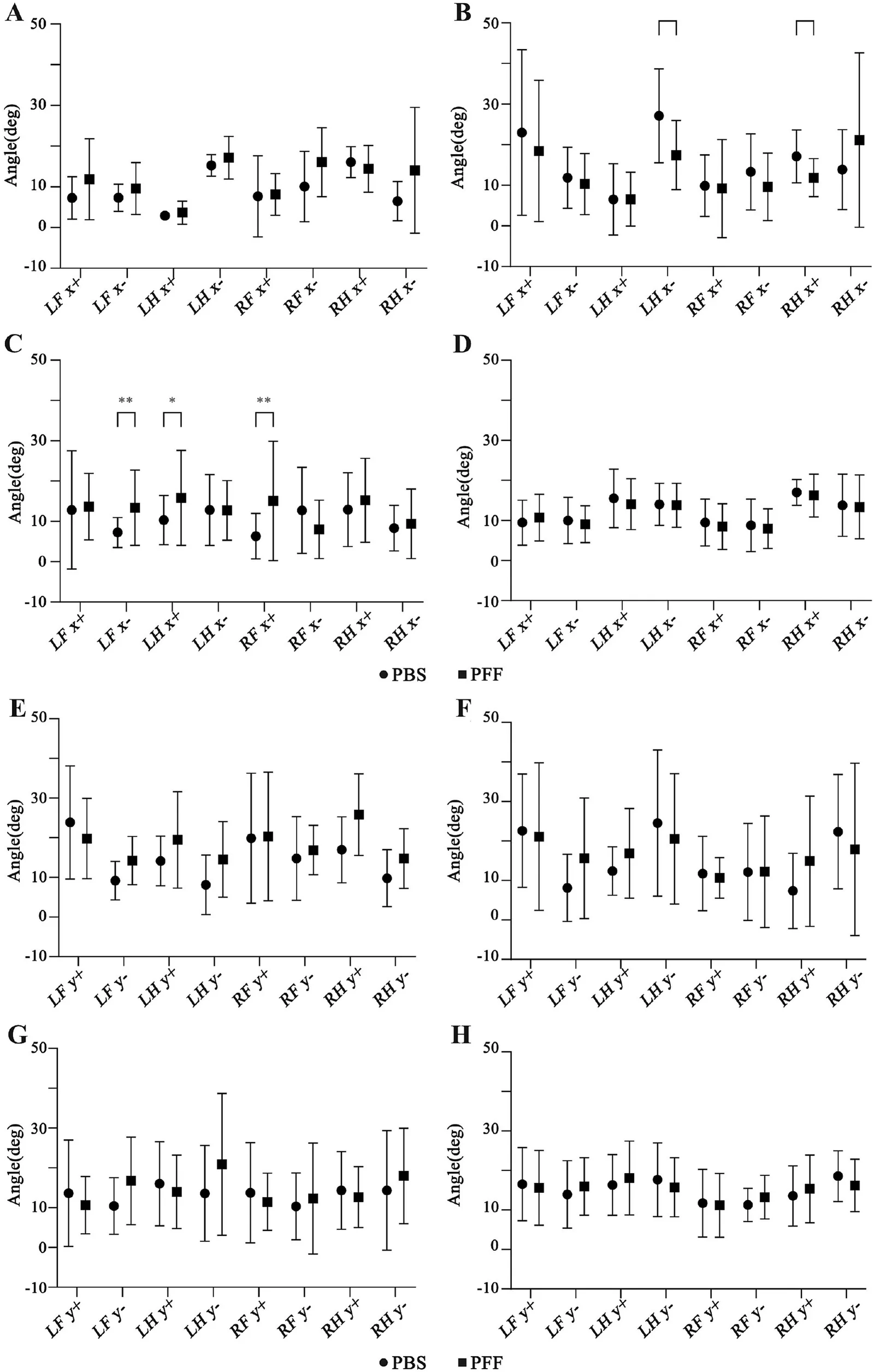
A-D: Maximum angles between limb ground reaction force vectors and the *Z*-axis in the X–Z plane at 0, 1, 3, and 6 months post-modeling. *E*-H: Maximum angles between limb GRF vectors and the Z-axis in the Y–Z plane at 0, 1, 3, and 6 months post-modeling.

## 4 Discussion

The present longitudinal analysis showed that biomechanical changes did not emerge simultaneously across all outcome measures. At 1-month post-inoculation, the maximum force-vector angle of the left hindlimb differed between PFF-injected and control mice, and a separate difference was observed in right forelimb GRF. GRF magnitude and temporal gait parameters did not show the same pattern at this time point. The force-vector angle may therefore represent a candidate biomechanical measure for longitudinal characterization in this model, although the present data do not establish it as a validated sensitive measure or an early diagnostic marker for human PD.

At 3-months post-inoculation, the biomechanical changes became more extensive. PFF-injected mice showed reduced left-side GRF, reductions in the right and left hindlimb GRF in the four-limb analysis, prolonged gait-cycle duration, prolonged stance-phase duration, and increased interlimb asymmetry. These findings may reflect altered weight distribution and impaired coordination during locomotion after unilateral striatal PFF inoculation. At 6 months, the reduction in left-side loading and the left-right GRF asymmetry persisted, whereas some between-group differences in gait duration and force-vector angles became less pronounced. The present measurements cannot determine whether this convergence resulted from compensatory adaptation, increased temporal variability, or a change in locomotor strategy. It is not interpreted as evidence of the recovery of motor function.

Previously published pathological findings from an independent cohort generated using the same PFF preparation procedure and a closely related unilateral intrastriatal paradigm provide context for the temporal pattern observed here. In that cohort, phosphorylated α-Syn was increased at 3 months, whereas further α-Syn accumulation, reduced TH expression, loss of TH-positive neurons, and motor deficits were observed at 6 months [28]. These findings are consistent with the expected pathological evolution of the model but do not establish a direct quantitative or causal relationship between pathological burden and the biomechanical measures reported here. In particular, no individual-level correlation between pathological measures and GRF or gait parameters was performed in the present study.

The present study also did not include direct correlation analyses with established behavioral assessments. Although the force-sensor array provides quantitative measures of limb loading, gait timing, and force-vector orientation, these biomechanical measures do not replace behavioral tests of motor coordination, balance, or spontaneous activity. They should therefore be regarded as complementary functional readouts. Future studies should obtain biomechanical and behavioral measurements from the same animals to determine whether individual-level changes in GRF and gait parameters are associated with established motor phenotypes.

The small sample size is another important limitation. Only three mice were included in each group. Although repeated walking trials improve measurement sampling, they do not increase the number of independent biological subjects. Thus, the present results may be influenced by inter-animal variability, and the statistical power to detect smaller effects is limited. Larger, independently replicated cohorts with a priori sample-size estimation will be required to confirm the reproducibility and robustness of the observed biomechanical changes.

The unilateral right-striatal PFF model provides a spatially defined perturbation and enables within-animal left–right biomechanical comparisons, which is useful for examining interlimb force redistribution. However, unilateral inoculation does not reproduce the bilateral and clinically heterogeneous nature of human PD. The left-sided GRF reductions observed in this study are contralateral to the right-striatal injection and may reflect both model-specific lateralization and disease-related motor dysfunction. Moreover, mouse and human locomotion differ substantially in body size, limb structure, gait organization, and locomotor strategy. Therefore, the biomechanical parameters identified here should be considered candidate measures for preclinical longitudinal characterization rather than directly translatable clinical biomarkers. Their relevance to human PD will require validation through larger animal cohorts, multimodal studies combining biomechanics, behavior, and pathology, and appropriately designed human studies.

## 5 Conclusions

Three-dimensional GRF analysis demonstrated time-dependent changes in locomotor biomechanics following unilateral intrastriatal α-Syn PFF inoculation. No significant between-group differences were detected at baseline (at 0 months). At 1 month, differences were observed in the maximum force-vector angle of the left hindlimb and right forelimb GRF. More extensive changes in left-side GRF, gait-cycle duration, and stance-phase duration were observed at 3 months. At 6 months, left–right GRF asymmetry persisted and increased, although some gait and force-vector measures showed smaller between-group differences.

Taken together, force-vector angle, left-side GRF, gait-cycle duration, stance-phase duration, and interlimb asymmetry may represent candidate biomechanical measures for exploratory longitudinal characterization of locomotor biomechanics in this mouse model. These findings should be considered preliminary and require validation in larger cohorts incorporating direct behavioral assessments and quantitative pathological measures.

## CRediT authorship contribution statement

**Mengyao Ning:** Writing – review & editing, Validation, Supervision, Formal analysis. **Jinpeng Li:** Writing – original draft, Visualization, Software. **Sheng Yang:** Resources, Methodology, Conceptualization. **Min Ye:** Supervision, Investigation. **Yi Fan:** Visualization, Resources, Investigation, Conceptualization. **Zhendong Dai:** Funding acquisition, Formal analysis, Conceptualization.

## Ethics statement

Supported by the Institutional Animal Care and Use Committee of 10.13039/501100007289Nanjing Medical University (IACUC-2103021).

## Ethics declaration

Animal subject: This study was conducted in accordance with the ARRIVE (Animal Research: Reporting of In Vivo Experiments) guidelines.This study was approved by the Experimental Animal Protection and Ethics Committee of Nanjing Medical University.(Approval No. IACUC-2103021).

## Funding

This research was funded by the National Natural Science Foundation of China Key Project (62233008).

## Declaration of competing interest

There are no conflicts to declare.
